# Magnesium film-over-nanospheres (FONs) for surface-enhanced Raman scattering

**DOI:** 10.1039/d5fd00120j

**Published:** 2026-01-23

**Authors:** Andrey Ten, Vladimir Lomonosov, Zeki Semih Pehlivan, Emilie Ringe

**Affiliations:** a Department of Materials Science and Metallurgy, University of Cambridge 27 Charles Babbage Road Cambridge CB3 0FS UK er407@cam.ac.uk; b Department of Earth Sciences, University of Cambridge Downing Street Cambridge CB2 3EQ UK

## Abstract

Understanding interfacial interactions at the native oxide surface of Mg is important for catalytic and biomedical applications. Recent advancements in plasmonic Mg research led to its use for surface-enhanced Raman scattering (SERS), a vibrational spectroscopy particularly sensitive to molecules bound to the enhancing substrate. Here, novel Mg film-over-nanospheres (FONs) are fabricated and used as SERS substrates to study molecular binding on Mg surfaces, augmenting previous results on 4-mercaptobenzoic acid (4-MBA) and 4-nitrothiophenol (4-NTP) by revealing the binding of 4-nitrophenol (4-NP) and 5,5′-dithiobis (2-nitrobenzoic acid) (DTNB). Through a systematic study of a total of 17 molecules with various functional groups, a p*K*_a_-related trend is unravelled: binding on natively oxidised Mg requires the adsorbate to dissociate and have a p*K*_a_ between ∼4.5 and 7.5, however diselenides and ditellurides do not appear to follow this trend. The results pave the way for chemical functionalisation of Mg surfaces for plasmonic and other applications.

## Abstract 2

To bind on natively oxidised Mg, adsorbates need a functional group with a p*K*_a_ between ∼4.5 and 7.5.

## Graphical abstract

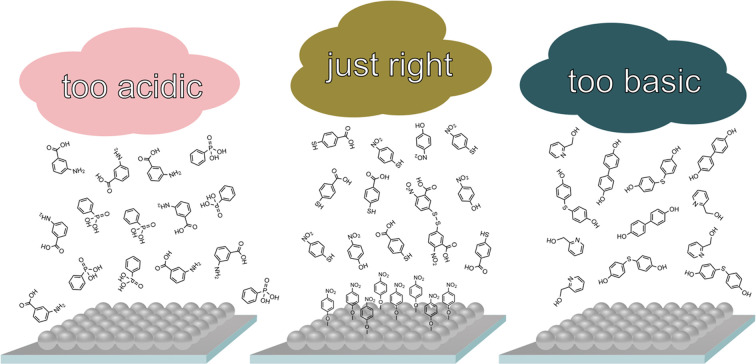

## Introduction

Understanding surface binding of chemical species at solid–gas and solid–liquid interfaces is important for a number of applications^1,2^ including catalytic transformations,^3,4^ electrochemistry,^5,6^ and biomedicine.^7,8^ Binding ability, strength, and orientation are indeed key factors affecting catalytic activity, electrochemical reactivity, and biological response. Better understanding of binding at interfaces can therefore assist in the design and analysis of such processes.

Vibrational spectroscopy is a fingerprinting tool that can identify molecules and their binding at the interface without the need for labels.^1,9^ It compares favourably to other techniques for its versatility of probing solid, liquid, and gaseous species and for the availability of reference data.^9^ The results can also be readily interpreted based on knowledge of molecular vibrations from group theory.^9^ Among several surface-sensitive vibrational spectroscopy techniques,^10,11^ this study focuses on surface-enhanced Raman scattering (SERS). SERS utilises the localised electric field from localised surface plasmon resonances (LSPRs).^12^ The Raman signal of molecules positioned in hot spots, the region of concentrated electric field, is significantly enhanced, in many cases by multiple orders of magnitude enabling sensitivity to single or a few molecules.^13^ Electromagnetic hot spots are formed on surfaces of plasmonic nanoparticles (NPs) at sharp tips or inter-particle gaps,^14^ or on surface features in fabricated structures such as film-over-nanosphere (FON) substrates.^15,16^ Other than the electromagnetic enhancement, the change in polarisability upon surface binding of the molecule also contributes to the enhancement; this phenomenon is part of the chemical (as opposed to electromagnetic) enhancement mechanism in SERS.^17^ Together, such enhancement enables the detection and identification of molecules at a surface, with the caveat that, according to Fang *et al.*, a majority (*e.g.*, around 85%) of the signal originates from a minority (*e.g.*, less than 6%) of molecules and thus the SERS signal may not be representative of the entire surface.^18^

SERS has previously helped to determine conformations of molecules on interfaces and to reveal the adsorption of molecules on plasmonic surfaces, not long after the technique’s discovery.^19^ Today, changes to Raman shifts and intensities are routinely tracked to determine the nature of adsorption, alongside computational techniques that complement experimental results.^20–23^ Spectral changes provide clues regarding molecular conformation, binding strength, and adsorbate-surface distance. However, SERS substrates are limited to very few plasmonic metals, constraining SERS interfacial studies to a narrow set of surfaces. The surface of plasmonic metals can be modified with thin layers, for instance through core–shell architectures, and expand the utility of SERS. However, any non-plasmonic layer distances the adsorbate from the metal surface and lowers the electromagnetic field enhancement.

Recent advances in sustainable and earth-abundant metals for plasmonics brought attention to Mg. Structural and optical properties of Mg-based structures have been characterised,^24,25^ confirming their plasmonic properties. Mg forms a native and self-limiting oxide layer that provides stability and opens opportunities for various applications.^26^ Indeed, colloidal Mg NPs have been successfully demonstrated as a SERS substrate, enhancing the signal of surface-bound 4-mercaptobenzoic acid (4-MBA) and 4-nitrothiophenol (4-NTP).^27^ This previous study suggested binding on the Mg NP surface through the thiol group, however, we could not rationalise the lack of binding *via* the carboxyl group in 4-MBA. Discussion with the SERS community highlighted the need for a systematic study on interfacial binding between Raman reporter molecules and the native Mg surface, leading to this paper. Here, we engineered, for the first time, a plasmonic Mg FON substrate, then used it to screen 17 molecules with a wide variety of functional groups, leading to a better understanding of binding on natively oxidised Mg surfaces.

## Methods

### Materials

Polystyrene (PS) nanospheres suspension in water (350 nm in diameter, 2.6% w/v) was purchased from PolySciences, Inc. Ti pellets (99.995%) and Mg pellets (99.95%) were purchased from Kurt J. Lesker and used as supplied. 4-MBA (99%), 4-NTP (80%), 4-nitrophenol (4-NP, 99%), 5,5′-dithiobis(2-nitrobenzoic acid) (DTNB, 99%), 4,4′-thiodiphenol (99%), 1-phenyl-2-pyrrolidinone (99%), diphenyl sulfide (98%), diphenyl diselenide (98%), 4-fluoroaniline (99%), diphenyl ditelluride (98%), 3-aminobenzoic acid (98%), 2-pyridinemethanol (98%), potassium thiocyanate (99.0%), triphenylphosphine (99%), ethanol and anhydrous isopropanol (IPA) were purchased from Sigma-Aldrich. Diethyl(4-nitrobenzyl)phosphonate (98%) was purchased from Thermo Fisher Scientific. 4,4′-dihydroxybiphenyl (99%) was purchased from Alpha Aesar. Phenylphosphonic acid (95%) was purchased from Fluorochem.

### Mg FON fabrication

Mg FONs were fabricated following the deposition conditions established previously.^28^ PS nanospheres (800 µL) were centrifuged (10 min, 10k rpm) and supernatant was removed. The nanospheres were resuspended in a 1 : 1 volume ratio mixture (600 µL) of deionised water (18.2 MΩ cm) and ethanol. The resulting nanosphere suspension was fed into a bath of deionised water through a partially immersed glass slide at 10 µL min^−1^ using a syringe pump, forming a monolayer of hexagonally close-packed nanospheres at the air–water interface. The resulting monolayers were collected onto 25 × 25 mm glass slides and left to dry in air.

Metal deposition by high vacuum thermal evaporation was performed using a CreaPhys GmbH single chamber deposition system positioned in a N_2_-filled glovebox. Ti and Mg were each placed on a boat-type metal evaporator and glass slides of PS nanosphere monolayers were attached to the substrate holder, before evacuating the chamber to high vacuum. Prior to the deposition, sacrificial Mg was evaporated to condition the chamber with closed shutters between the source and the sample. The chamber pressure dropped as Mg captured the remaining oxygen and water. Once the pressure stabilised at around 1 × 10^−6^ mbar, Ti was evaporated (0.1 Å s^−1^) to 5 nm as an adhesion layer, before evaporating Mg (1.5 Å s^−1^) to 100 nm. The evaporation rate was monitored using a quartz crystal microbalance.

### Mg FON functionalisation

0.01 M solutions of each Raman reporter molecule (4-MBA, 4-NTP, 4-NP, DTNB, 4,4′-thiodiphenol, 1-phenyl-2-pyrrolidinone, diphenyl sulfide, diphenyl diselenide, 4-fluoroaniline, diphenyl ditelluride, 3-aminobenzoic acid, 2-pyridinemethanol, potassium thiocyanate, triphenylphosphine, diethyl(4-nitrobenzyl)phosphonate, 4,4′-dihydroxybiphenyl, and phenylphosphonic acid) were prepared in IPA. For each molecule, the solution was drop-casted onto an individual Mg FON and dried in air. FONs were then each rinsed with IPA to remove unbound molecules and dried in air.

### Optical characterisation

Ultraviolet-visible-near infrared (UV-vis-NIR) spectroscopy was conducted using a Thermo Fisher Evolution 220 UV-visible spectrophotometer. Absorption spectra were collected in transmittance mode. Reflectance spectrum of Mg FON was collected using an ISA-220 integrating sphere accessory, equipped with an 8° wedge plate, in reflectance mode. The Spectralon disc was used as the reflectance reference material for baseline correction.

Scanning electron microscopy (SEM) was performed using a ZEISS GeminiSEM operated at 5.00 kV. Images were collected using an Everhart–Thornley detector for secondary electron imaging.

### SERS and Raman measurements

SERS and Raman spectroscopy were performed using a HORIBA Jobin Yvon LabRam300 confocal Raman system, equipped with an Olympus BXFM-ILHS microscope with motorized *z*-axis of freedom and a motorised *x*,*y*-adjustable stage. Measurements were performed using a continuous wave 532 nm Nd:YAG laser operated at 20 mW output power attenuated between 1 and 100% power using optical density filters, an Olympus LMPlanFl 50×/0.50 objective, 600 g mm^−1^ grating, 100 µm slit, 400 µm hole, and LabSpec 6 Spectroscopy Suite software. For each sample, data were collected across 20 different regions and summed. The beam diameter at sample was 2.5 µm. The laser power at sample was 85.7, 717, 1870, 3840, and 7270 µW at 1, 10, 25, 50, and 100% attenuation, respectively, as measured using a Thorlabs slim Si sensor (400–1100 nm, 500 pW–500 mW) attached to a Thorlabs PM100D digital console. Data analysis was performed using Origin Pro. Intensity values were converted to analogue to digital converter units (ADU) during the analysis, by dividing signal intensity with laser power at sample and integration time.

## Results and discussion

Mg forms a native, self-limiting oxide layer. This ∼7 nm surface layer^28^ protects the underlying metallic structure from further oxidation, for months in ambient air, and during heating up to 400 °C.^26^ This protective outer layer contains crystalline MgO, as previously observed by X-ray diffraction and high-resolution transmission electron microscopy,^24,26^ but its surface is likely readily hydroxylated to Mg(OH)_2_. The potential to tailor the surface chemistry, from oxide to hydroxide, offers opportunities in, *e.g.*, catalysis and chemical functionalisation. However, the nature of such dynamic surface remains challenging to characterise, and little is known about the binding of molecules on this oxidised layer, limiting the current scope of surface modification.

**Fig. 1:**
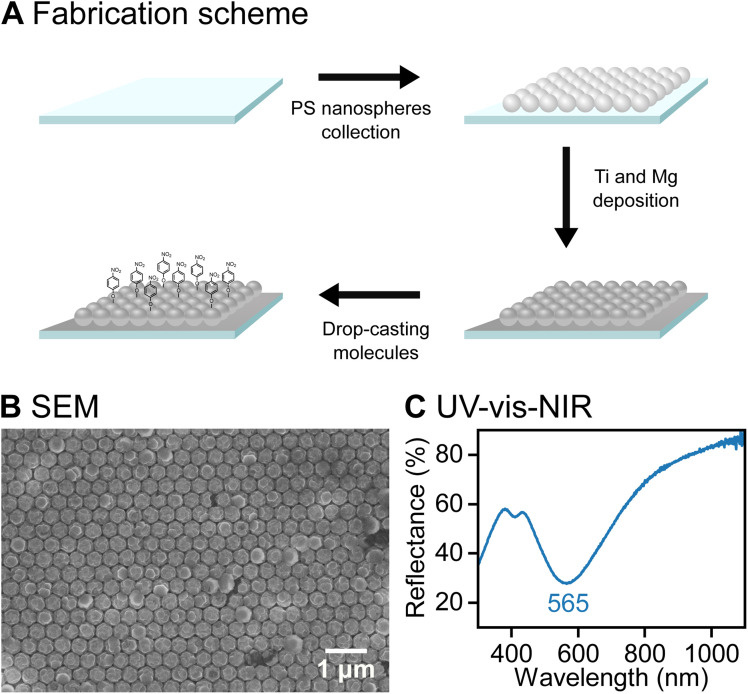
Plasmonic Mg FONs. (A) Schematic of Mg FON fabrication and functionalisation with Raman reporter molecules. (B) SEM and (C) UV-vis-NIR reflection spectrum of a fabricated Mg FON. The inverse peak in the reflection spectrum corresponds to the LSPR.

FON, a well-established SERS substrate geometry for plasmonic metals,^16,29^ was selected here to study molecular binding to Mg surface. FON offers benefits such as the ease of tuning LSPR energy by changing the size of nanospheres, reproducibility across large areas, and a surface free from surfactants or ligands. In this paper, the fabrication of Mg FONs was achieved for the first time. Mg (100 nm thickness) was deposited on a close-packed array of 350 nm PS nanospheres (Fig. 1A, B and S1), using deposition conditions similar to those we used previously to create Mg triangular arrays.^28^ A Ti layer (5 nm thickness) was used as an adhesion layer between the PS and Mg. An oxidised Mg surface layer was formed spontaneously upon removal of the sample from the deposition chamber and exposure to ambient air. The resulting Mg FON had LSPR energy at 565 nm as observed by UV-vis-NIR spectroscopy (Fig. 1C), well-aligned to the SERS excitation of 532 nm.

**Fig. 2:**
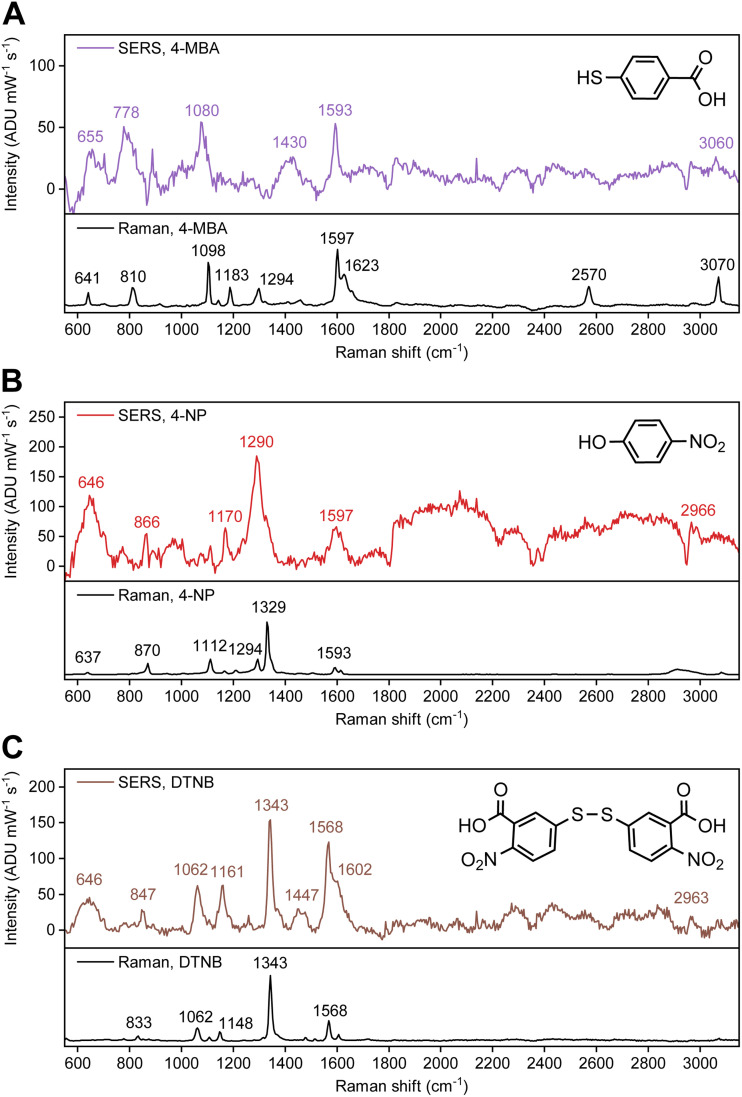
SERS spectra of Raman reporter molecules on Mg FONs at 532 nm laser excitation. Spectra of (A) 4-MBA, (B) 4-NP, and (C) DTNB. Normal Raman spectra are presented as a reference, with the 4-MBA spectrum reproduced from ref. 27. Spectra prior to background subtraction are presented in Fig. S2.

SERS spectra from Mg FON and colloidal Mg NP substrates were first compared using the (same) Raman reporter molecule, 4-MBA. The 4-MBA SERS spectrum from Mg FONs (Fig. 2A and S2) contained peaks at 1080 and 1593 cm^−1^, corresponding to D_6_ and D_3_ modes, respectively. This labelling follows the Mulliken scheme for *para*-disubstituted benzene molecules with *C*_2v_ symmetry.^30,31^ The peak at 1430 cm^−1^, assigned to the carboxylate anion stretching mode, was also observed. These features in the spectrum are consistent with those seen from 4-MBA on colloidal Mg NP substrates as well as on other plasmonic materials,^32^ indicating successful Raman signal enhancement of surface-bound 4-MBA. The absence of 4-MBA signal on flat Mg films (Fig. S3) further confirms the field enhancement property of Mg FON substrates. The 4-MBA SERS spectrum did not contain characteristic peaks from PS nanospheres (Fig. S3), however, a large feature near 1000 cm^−1^ in Mg FONs, tentatively attributed to Mg due to its intensity being correlated with the Mg thickness, was present in the spectrum and required careful background subtraction. The SERS enhancement factor (EF) was assessed using the 4-MBA signal as described in the SI. The calculated enhancement factor was 8, which is roughly an order of magnitude lower than that observed with colloidal Mg NP substrates using the same Raman reporter molecule.^27^

The SERS-active Mg FON substrate was used as a tool to explore molecular binding on an Mg surface exposed to air (thus covered by a native, protective, and thin oxidised layer). A range of molecules with different functional groups with different electron affinity, were tested for their binding to this surface. Mg FON substrates were functionalised by drop-casting 0.01 M solutions of Raman reporter molecules in IPA, followed by drying. The substrates were then thoroughly rinsed with IPA, ensuring only bound molecules remained. More concentrated solutions (0.1 M) of some molecules, including 4-NTP and 4,4′-thiodiphenol, resulted in the Mg film detaching from the polystyrene beads, and thus were not used further. Overnight incubation of the substrates in IPA solutions did not further increase the surface coverage of the Raman reporters, as indicated by their SERS intensity.

Using SERS on Mg FON substrates, two new findings were unravelled: 4-NP and DTNB both bind to the natively oxidised surface of Mg. The spectrum of 4-NP (Fig. 2B and S2) contained four distinguishable peaks at the energies expected from SERS studies on other substrates.^33–36^ The peaks at 1170 and 1597 cm^−1^ are assigned to D_6_ and D_3_ modes, respectively,^33,34,36–38^ again following the Mulliken scheme under the assumption that 4-NP binds *via* either the nitro or the hydroxyl group (resulting in *C*_2v_ symmetry). The band at 866 cm^−1^ is a combination of the D_16_ mode and the NO_2_ bending mode,^33,36,38^ while the strong peak at 1290 cm^−1^ is attributed to the NO_2_ stretching mode.^34,36,38^ The position of the NO_2_ stretching mode indicates the presence of 4-nitrophenolate (4-NP^−^), the anionic form of 4-NP previously reported for Ag film^34^ and hydrosol^36^ substrates. The NO_2_ stretching mode of a neutral molecule is expected near 1330 cm^−1^ as observed in the normal Raman spectrum (Fig. 2B). The 4-NP dissociation is also observed in the Mg NP colloid, supporting its presence in the SERS experiments. Specifically, when a solution of 4-NP in IPA was mixed with colloidal Mg NPs, it turned yellow, with the absorption peak red-shifting from 310 to 410 nm (ref. 38) (Fig. S4) consistent with 4-NP^−^ formation. Note that, 4-NP dimerisation^33^ was not observed in the current study with Mg.

Determining the binding site and orientation of 4-NP^−^ on Mg surfaces from the SERS spectrum alone is impeded by to the lack of recognised reference data relating its spectrum with its binding configuration. Indeed, the binding site of the molecule on other surfaces, mostly Ag, is debated in the literature, with contradictory surface-enhanced vibrational spectroscopy studies suggesting vertical binding through either the hydroxyl O atom^39,40^ or the NO_2_ group,^37^ or horizontal binding through both functional groups.^34^

DTNB is a common Raman reporter molecule on Au surfaces and binds *via* the thiol group following the cleavage of the S–S bond.^41–43^ Put another way, it is essentially two individual 5-mercapto-2-nitrobenzoic acid (TNB) molecules binding on Au. The spectrum of DTNB on Mg FON (Fig. 2C and S2) contained peaks consistent to those reported for Au. The most intense peak was observed at 1343 cm^−1^, corresponding to the NO_2_ stretching mode.^41–44^ The peaks at 857, 1062, and 1568 cm^−1^ are the TNB-equivalent of D_16_, D_6_, and D_3_ modes, respectively.^41–44^ As for 4-NP, the 857 cm^−1^ peak contains a contribution from the NO_2_ bending mode.^43^ The main difference between normal Raman and SERS of DTNB on Mg FON was the lower relative peak intensity between the NO_2_ stretching mode at 1343 cm^−1^ and the other peaks in SERS. Otherwise, the differences in peak positions were not significant, as expected from previous Au-based studies.^41^ Despite these spectral similarities to literature and our previous results of successful thiol binding on Mg NPs,^27^ the lack of spectral differences between DTNB and TNB prevents us from confirming that DTNB binds on Mg as individual TNBs *via* S.

**Fig. 3:**
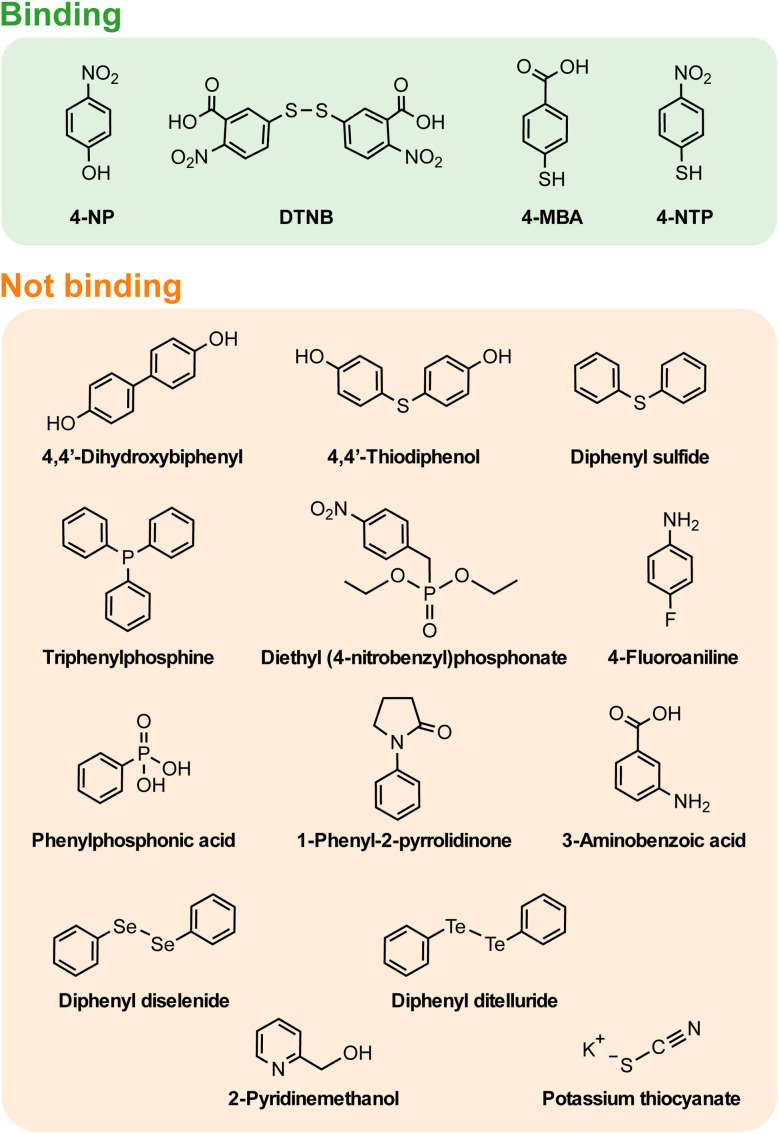
Molecules classified by their binding to the Mg surface. Classifications were made based on the presence (binding) or lack of (not binding) SERS signal on Mg FON substrates. 4-NTP was classified as binding based on the previous study with colloidal Mg NPs.^27^

**Fig. 4:**
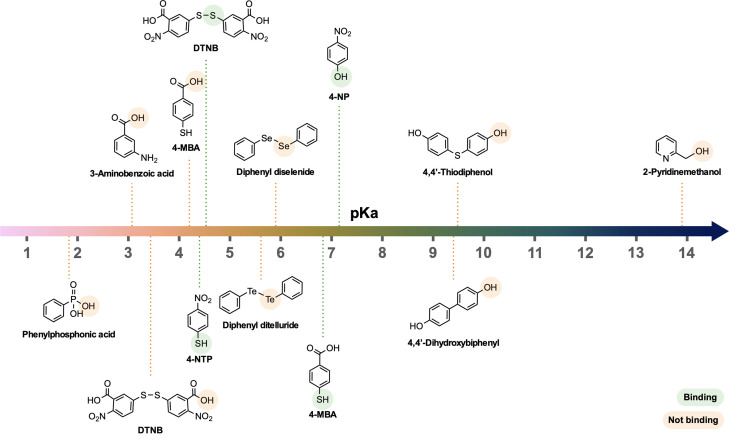
Functional groups of molecules tested using SERS, on Mg FONs organised by their p*K*_a_ values. The respective functional groups are highlighted, with green indicating binding and orange indicating lack of binding to Mg surface, based on the SERS spectra.

While both 4-NP and DTNB SERS spectra provide insight on their binding, further understanding can be gained by investigating the molecules that could not be detected in SERS, due to poor binding to the natively oxidised Mg surface. Fig. 3 shows the 17 molecules analysed using SERS, on Mg FONs, including those with signal too low to detect, suggesting a lack of strong binding. Such molecules include amines, alcohols, phosphonates, and chalcogenides. Given that the SERS spectra of 4-MBA, 4-NP, and 4-NTP ^27^ on Mg consistently showed the presence of anionic forms, the molecule dissociation was predicted to influence binding. The molecules tested for SERS, were organised by their p*K*_a_ values to rank their acidity (Fig. 4). Note, basic molecules, such as 4-fluoroaninline with an amine group, did not bind to Mg due to the basic nature of the surface oxide layer.

Molecules which bind to the Mg surface had aqueous p*K*_a_ values between 4.5 and 7.5, although not all molecules in this range could be detected. The thiol group of 4-MBA, through which the molecule binds,^27^ has a p*K*_a_ of 6.8.^45^ Similarly, the hydroxyl group p*K*_a_ of 4-NP is 7.15.^46^ The acidity of DTNB formed upon heterolytic S–S bond cleavage can be deduced from the p*K*_a_ of TNB, whose thiol p*K*_a_ is 4.53.^47^ Note that the thiolate cleavage may also be homolytic,^48^ forming two radicals.

None of the molecules with p*K*_a_ values lower than 4.5 bound to the Mg FON surface. Both 4-MBA and DTNB additionally have carboxyl groups whose p*K*_a_ is around 4.2 in 4-MBA (p*K*_a_ of benzoic acid = 4.20),^49^ and under 3.5 for DTNB (p*K*_a_ of 3-nitrobenzoic acid = 3.45).^49^ However, the carboxyl group was not the binding moiety for 4-MBA. Similarly, phenylphosphonic acid with phosphonate group p*K*_a_ of 1.83,^50^ and 3-aminobenzoic acid with a carboxyl group p*K*_a_ of 3.07 (ref. 51) did not result in sufficient SERS signal, implying the lack of surface binding.

On the other hand, all analysed Raman reporter molecules with p*K*_a_ values higher than 7.5 also failed to bind on Mg FONs. Hydroxyl group p*K*_a_ values of many molecules fall in this range, with 4,4′-dihydroxybiphenyl at 9.4 and 14.1,^52^ 4,4′-thiodiphenol near 9.5 (p*K*_a_ of 4-(methylthio)phenol = 9.47),^53^ and 2-pyridinemethanol around 13.9 (p*K*_a_ of choline = 13.9),^54^ all of which did not bind on Mg.

The observed p*K*_a_ dependence can be rationalised as follows. For molecules with a high p*K*_a_, there is a lack of dissociation and thus no binding to the Mg surface. In contrast, the interaction of Mg^2+^ with a weak conjugate base formed from molecules with low p*K*_a_, is not sufficient to bind and remain on the Mg surface.

Chalcogenides other than S did not bind to Mg FONs, despite their p*K*_a_s being in the previously described range, pointing to a more complex picture for related functional groups. The ionic strength of phenyl selenide was estimated from the p*K*_a_ of selenophenol which has a p*K*_a_ of 5.90.^55,56^ The p*K*_a_ of tellurophenol is not known but is expected to be lower than 5.90, following the trend in chalcogenides.^55^ The lack of binding of Se and Te is as yet unexplained, although it may be caused by the different splitting pattern of the chalcogen–chalcogen bond, or the higher polarisability of Se and Te compared to S.

## Conclusions

Surface binding properties on Mg have been studied by examining SERS spectra. A surfactant-free, plasmonic Mg surface was fabricated in the form of film-over-nanospheres (FON), which enabled the use of SERS to probe the surface. A range of 17 molecules with different functional groups were deposited onto the natively oxidised surface of Mg and their binding was assessed with SERS. The spectra revealed the binding of 4-NP and DTNB, which add to the previously known 4-MBA and 4-NTP. Assessment of molecules based on their binding properties revealed a correlation between binding and p*K*_a_ values, with only those between 4.5 and 7.5 adsorbing on the surface. These findings open the door for rationally functionalising Mg, a surface which is of interest in catalytic and biomedical processes.

## Author contributions

Andrey Ten: conceptualization, data curation, formal analysis, investigation, methodology, project administration, validation, visualization, writing – original draft, writing – review & editing. Vladimir Lomonosov: conceptualization, investigation, methodology, writing – review & editing. Zeki Semih Pehlivan: conceptualization, investigation, methodology. Emilie Ringe: conceptualization, funding acquisition, project administration, resources, supervision, writing – original draft, writing – review & editing.

## Conflicts of interest

There are no conflicts of interest to declare.

## Supplementary Material

FD-265-D5FD00120J-s001

## Data Availability

Supplementary information (SI): additional analytical details, SEM images, SERS spectra prior to background subtraction, Raman spectra, and UV-vis-NIR spectra. See DOI: https://doi.org/10.1039/d5fd00120j.
