# Supplementary material for: Magnesium film-over-nanospheres (FONs) for surface-enhanced Raman scattering
Source: Faraday Discuss. 2026 Jan 23. Online ahead of print. doi: 10.1039/d5fd00120j (PMC13127358; doi:10.1039/d5fd00120j)
Supplement: FD-OLF-D5FD00120J-s001 [file FD-OLF-D5FD00120J-s001.pdf]

Supplementary Information (SI) for Faraday Discussions.
This journal is © The Royal Society of Chemistry 2026

Electronic Supplementary Information

Magnesium film-over-nanospheres (FONs) for surface-enhanced Raman scattering

Andrey Ten,[a,b] Vladimir Lomonosov,[a,b] Zeki Semih Pehlivan[a,b] and Emilie Ringe[a,b]*

a. Department of Materials Science and Metallurgy, University of Cambridge, 27 Charles Babbage Road, Cambridge CB3 0FS, United Kingdom
b. Department of Earth Sciences, University of Cambridge, Downing Street, Cambridge CB2 3EQ, United Kingdom

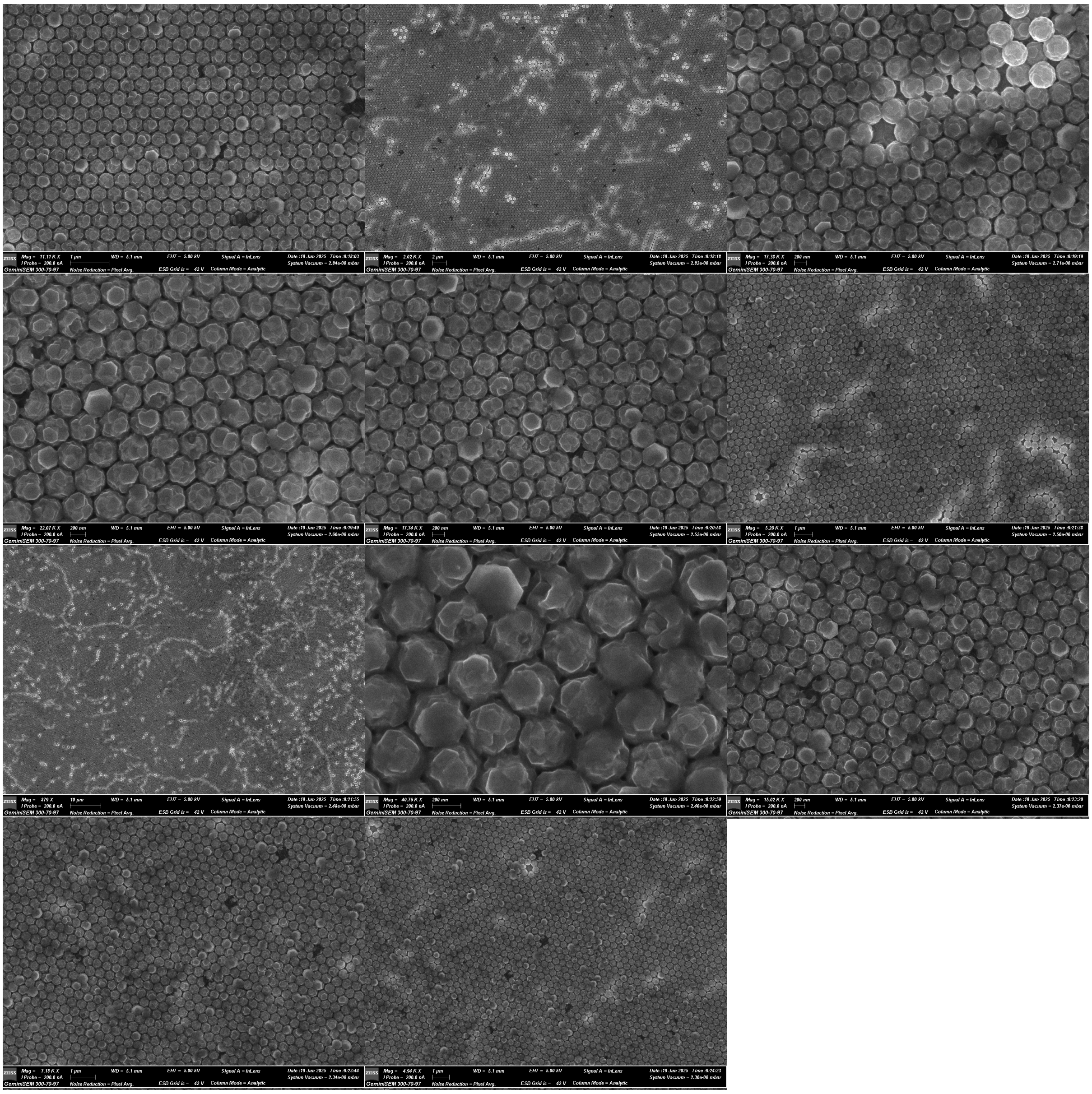

Figure S1. Additional SEM images of Mg FONs.

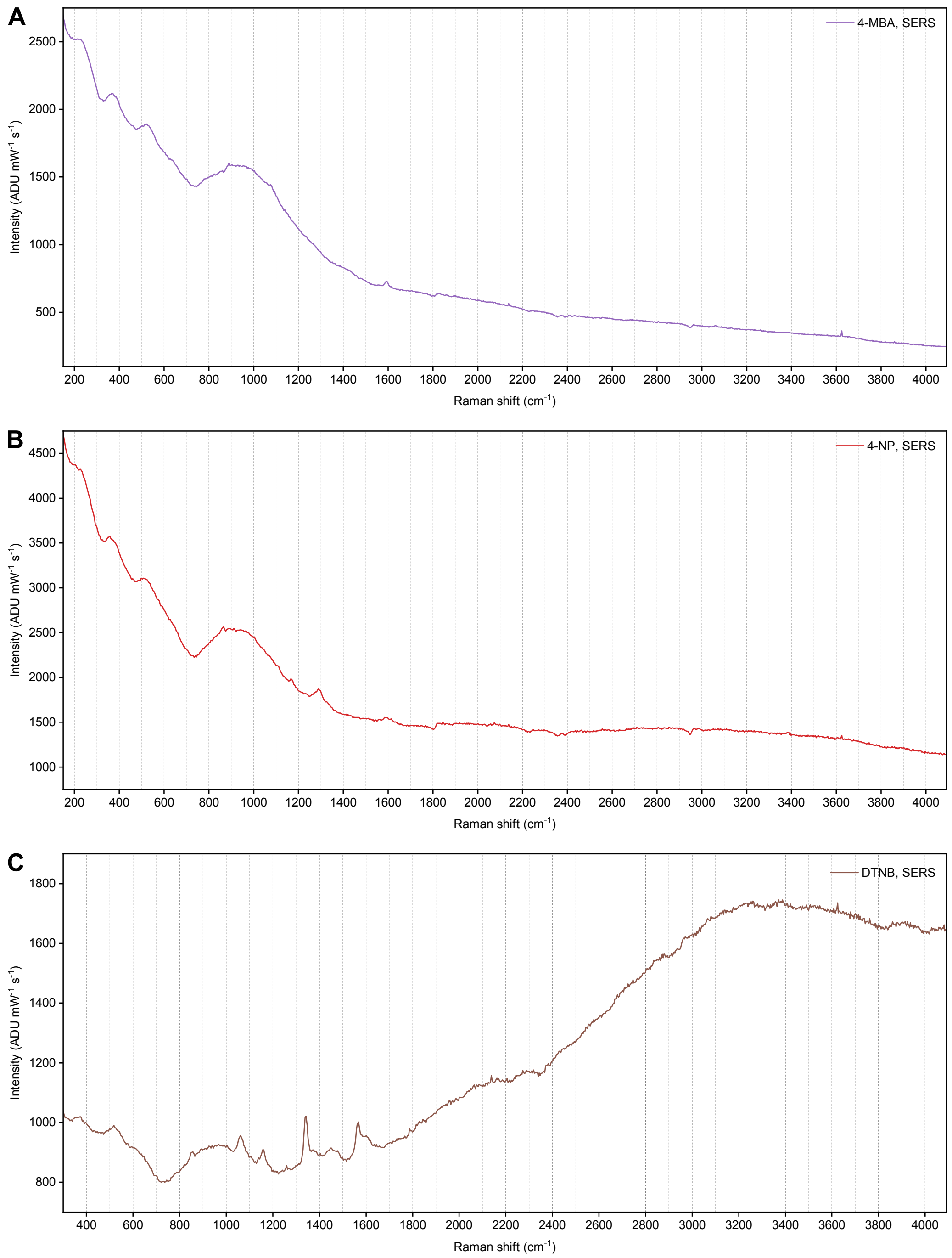

Figure S2. Full SERS spectra of Raman reporter molecules prior to background subtraction, for (A) 4-MBA, (B) 4-NP, and (C) DTNB.

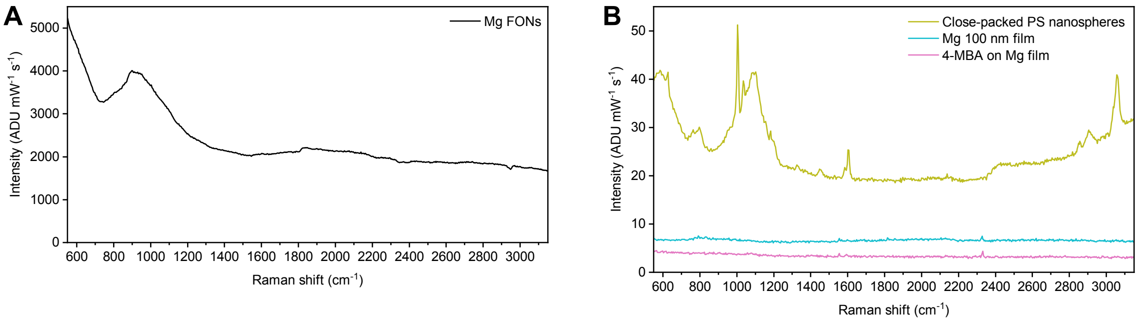

Figure S3. Additional Raman spectra collected as a control. (A) Spectrum of Mg FONs without any molecules deposited. (B) Spectrum of 100 nm (flat) Mg film deposited on glass on top of a 5 nm Ti adhesion layer, of flat Mg film with 4-MBA deposited on top, and of close-packed PS nanospheres on glass.

SERS enhancement factor (EF) calculation

The SERS EF of Mg FON was calculated using the following equation:[1]

$$\mathrm{EF} = \frac{A_{\mathrm{SERS}}}{A_{\mathrm{Raman}}}\frac{N_{\mathrm{Vol}}}{N_{\mathrm{Surf}}}$$

Where A_{SERS} and A_{Raman} are the areas under the peak of the D_3 mode around 1580 cm^{-1}, and N_{Surf} and N_{Vol} are the number of Raman reporter molecules for SERS and Raman, respectively. The D_3 mode was chosen as it did not overlap with any IPA signal in 0.1 M 4-MBA solution in IPA, which was used as a Raman reference. The scattering cross section in solution was estimated to be a sphere with a diameter of 2.5 μm, and was multiplied by the concentration of the solution to give 4.93×10^8 molecules for N_{Vol}. For N_{Surf}, the surface of a FON was estimated to be a collection of hemispheres, which increases the surface area by a factor of 2 compared to a flat surface. Dividing the total FON surface area under a 2.5 μm beam by a thiol footprint[2] of 0.22 nm^2 gives 1.78×10^8 molecules, although this is likely an overestimate. With area under the peak being 731 and 262 ADU mW^{-1} s^{-1} for SERS and Raman, respectively, the final EF is calculated to be 8, a lower bound due to the conservative assumption of coverage density.

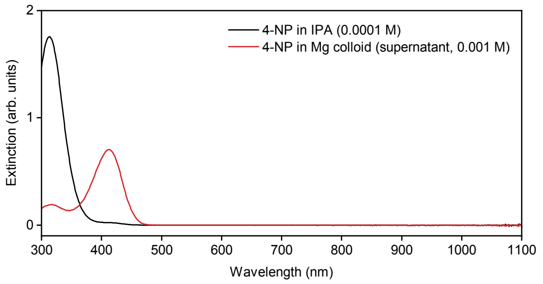

Figure S4. 4-NP dissociates to 4-NP⁻ upon mixture with Mg NPs. UV-vis-NIR spectra of 4-NP (black) before and (red) after mixing with Mg NPs.

References

1. E. C. Le Ru and B. Auguié, *ACS Nano*, 2024, **18**, 9773–9783.

2. C. G. Khoury and T. Vo-Dinh, *J. Phys. Chem. C*, 2008, **112**, 18849–18859.
